# Personalized Antiplatelet Therapy Based on CYP2C19 Genotypes in Chinese ACS Patients Undergoing PCI: A Randomized Controlled Trial

**DOI:** 10.3389/fcvm.2021.676954

**Published:** 2021-06-16

**Authors:** Xiujin Shi, Yunnan Zhang, Yi Zhang, Ru Zhang, Baidi Lin, Jialun Han, Wenzheng Li, Zhenwei Fang, Jialin Yan, Yifan Wang, Ze Zheng, Yuan Lv, Yang Lin

**Affiliations:** ^1^Department of Pharmacy, Beijing Anzhen Hospital, Capital Medical University, Beijing, China; ^2^School of Pharmaceutical Sciences, Capital Medical University, Beijing, China; ^3^Department of Cardiology, Beijing Anzhen Hospital, Capital Medical University, Beijing, China

**Keywords:** CYP2C19, personalized antiplatelet therapy, ticagrelor, clopidogrel, acute coronary syndrome

## Abstract

**Background:** The clinical benefits of cytochrome P450 (CYP) 2C19 genotype-guided antiplatelet therapy in Asians remain unclear. In this study, we aimed to investigate the clinical outcomes of pharmacogenomic antiplatelet therapy in Chinese patients.

**Methods:** Patients with acute coronary syndrome planning to undergo percutaneous coronary intervention were eligible for this study and were randomly divided into a genotype-guided treatment (GT) group and routine treatment (RT) group, with a ratio of 2:1. Patients in the GT group underwent CYP2C19 genotyping (^*^2 and ^*^3 alleles), and the results were considered in selecting P2Y_12_ receptor inhibitors. Patients in the RT group were treated with P2Y_12_ receptor inhibitors according to their clinical characteristics. The primary endpoint was a composite of major adverse cardiovascular or cerebrovascular events (MACCE). The secondary endpoint was significant bleeding events.

**Results:** Finally, 301 patients were enrolled; 75.1% were men and the mean age was 59.7 ± 9.8 years. In total, 281 patients completed the follow-up procedure. The primary endpoint occurred in 16 patients, 6 patients in the GT group and 10 in the RT group. The GT group showed lower MACCE rates than the RT group (6/189 vs. 10/92, 3.2 vs. 10.9%, hazard ratio: 0.281, 95% confidence interval: 0.102–0.773, *P* = 0.009). There was no statistically difference in significant bleeding events between the GT and RT groups (4.2 vs. 3.3%, hazard ratio: 1.315, 95% confidence interval: 0.349–4.956, *P* = 0.685).

**Conclusion:** Personalized antiplatelet therapy that is based on CYP2C19 genotypes could decrease MACCE within a 12-month period in Chinese patients with acute coronary syndrome undergoing percutaneous coronary intervention.

**Clinical Trial Registration:** http://www.chictr.org.cn, identifier: ChiCTR2000034352.

## Introduction

Dual antiplatelet therapy (DAPT), the combination of aspirin and a P2Y_12_ receptor inhibitor, is recommended for patients with acute coronary syndrome (ACS), especially for those undergoing percutaneous coronary intervention (PCI) (1, 2). Substantial evidence has confirmed that DAPT can improve the cardiovascular outcomes of patients with ACS and reduce the risk of all-cause death (3, 4). Clopidogrel is the most widely used P2Y_12_ receptor inhibitor in clinical settings (5). However, clopidogrel is a prodrug requiring biotransformation by cytochrome P450 (CYP) 2C19, which leads to substantial variability in patient responses. Patients carrying CYP2C19 loss-of-function (LOF) alleles show absent or reduced activity in clopidogrel metabolism (6), which contributes to failed antiplatelet therapy during clopidogrel treatment and may even lead to ischemia (7). Ticagrelor is not influenced by CYP2C19 gene polymorphism (8). It has been reported that ticagrelor possesses more potent antiplatelet efficacy than clopidogrel (9) and is recommended as a front-line P2Y_12_ inhibitor in the international guidelines (10, 11). However, the higher bleeding risk and adverse effects such as dyspnea limit the widespread use ticagrelor and lead to pre-mature cessation of treatment (12). As such, prescribers are still unclear on how to balance ischemic risk and bleeding risk.

CYP2C19 genetic testing has been confirmed to help in selecting P2Y_12_ inhibitors, thereby personalizing DAPT, which then leads to better outcomes in patients with ACS in comparison with routine therapy (13, 14). However, international guidelines regarding this novel strategy are varied (15, 16), and until now, CYP2C19 genotype-guided personalized antiplatelet therapy has not been widely accepted in the clinic. However, most studies used to inform these guidelines included Caucasian patients (14). The CYP2C19 LOF allele frequency among East Asian patients is considerably higher than in other populations (17); however, the clinical benefits of personalized antiplatelet therapy that is based on CYP2C19 genotypes in Asian patients still lacks clear evidence, especially over a long-term follow-up period.

Consequently, we conducted the present open-label, randomized clinical trial to identify whether the use of personalized DAPT according to CYP2C19 genotypes could lead to better outcomes within a 12-month period in Chinese patients with ACS undergoing PCI.

## Methods

### Patients

This single-center, open-label, randomized trial was performed at Beijing Anzhen Hospital [Chinese Clinical Trial Registry (http://www.chictr.org.cn), ChiCTR2000034352]. The research protocol was approved by the ethics committee of Anzhen Hospital. All patients provided informed consent prior to study inclusion.

Patients were eligible for this study if they were age 18 years or older, diagnosed with ST-elevation myocardial infarction (STEMI), non-STEMI, or unstable angina and required revascularization by PCI. The definitions of ACS were as follows. (1) STEMI: ischemic symptoms and persistent ST-segment elevation on electrocardiogram (ECG) or new left bundle branch block with elevated biomarkers of myocardial necrosis (creatine kinase-MB or troponin > upper reference limit); (2) non-STEMI: elevated biomarkers of myocardial necrosis with transient ST-segment elevation or depression or T-wave changes consistent with myocardial ischemia; and (3) unstable angina: recurrent ischemic chest pain at rest or on minimal exertion and new or worsening ST or T-wave changes in at least two leads in the absence of elevated biomarkers of myocardial necrosis (18). The diagnosis and PCI indication were in line with the Chinese Guidelines for Percutaneous Coronary Intervention (2016) (19). The exclusion criteria were: (1) contraindicated or allergic to ticagrelor or clopidogrel; (2) requiring long-term use of anticoagulation treatment with warfarin or other therapy; (3) life expectancy < 1 year; (4) intracranial bleeding history or high risk of major bleeding; (5) requiring dialysis; and (6) pregnant and lactating women.

### Randomization

Patients providing informed consent were randomly divided into a genotype-guided treatment (GT) group and a routine treatment (RT) group prior to the PCI procedure. Computer random number generation was used to assign the patients, with an allocation ratio of 2:1 (GT:RT), which was carried out by an independent investigator using blocked randomization. The block sizes were 6, 9, and 12. After the randomization sequence was generated, it was sent to cardiologists in sealed envelopes. This was an open-label trial, so no masking of the treatment assignment was used.

### CYP2C19 Genotyping

Patients in the GT group underwent CYP2C19 genotyping (^*^2, ^*^3 alleles) as soon as possible after randomization, and the results were returned within 48 h after the collection of blood samples. In brief, the processes of CYP2C19 genotyping were as follows. Genomic DNA was extracted from leucocytes of peripheral blood and stored in 3 mL ethylenediaminetetraacetic acid-anticoagulated vacuum tubes. CYP2C19 genotypes were determined using fluorescence *in situ* hybridization (TL988A, Xi'an TianLong) including the following variant alleles: CYP2C19*2 (rs4244285) and CYP2C19*3 (rs4986893). The whole process was performed according to the manufacturer's instructions.

In keeping with the Clinical Pharmacogenetics Implementation Consortium (20), we classified the CYP2C19 genotyping results into LOF allele carriers (^*^1/^*^2, ^*^1/^*^3, ^*^2/^*^2, ^*^2/^*^3, ^*^3/^*^3) and non-carriers of LOF alleles (^*^1/^*^1).

### P2Y_12_ Inhibitor Treatment

All patients were treated with the loading dose of ticagrelor 180 mg or clopidogrel 300 mg prior to PCI, then treated with the maintenance dose, ticagrelor 90 mg twice a day or clopidogrel 75 mg once a day (19, 21). The initial selection of P2Y_12_ receptor inhibitors was made according to patients' clinical characteristics, including age, body mass index, medical history, bleeding risk, and ischemic risk. The selection of P2Y_12_ inhibitors in the GT group was reconsidered by cardiologists after obtaining the genetic test results. CYP2C19 LOF allele carriers were recommended treatment with ticagrelor, but the ultimate decision was made after taking into account the clinical characteristics. Prescription of P2Y_12_ inhibitors was at the discretion of cardiologists. Any changes in P2Y_12_ receptor inhibitors after discharge were recorded. All patients took 100 mg aspirin daily.

### Study Endpoints

The primary endpoint was a composite of major adverse cardiovascular or cerebrovascular events (MACCE), defined as the composite endpoint of all-cause death, myocardial infarction, stroke, urgent coronary revascularization, and stent thrombosis within 12 months after the index PCI. Urgent coronary revascularization was defined as unplanned coronary revascularization caused by ACS.

The secondary endpoint was significant bleeding events. In keeping with the Bleeding Academic Research Consortium (BARC) (22), we defined BARC class 2 or higher as a significant bleeding event. All events were verified by the Independent Response Evaluation Committee (IREC) consisting of two cardiologists and two pharmacists who were blinded to the patient groups and the P2Y_12_ receptor inhibitors received by patients.

### Follow-Up

The time of the index PCI was considered time 0. At 6 and 12 months, patients were interviewed *via* office visits, telephone, or WeChat. The follow-up procedure was performed by trained pharmacists who were unaware of the group assignments. Information on adverse and endpoint events and compliance with treatment were obtained at every interview.

### Statistical Analysis

The sample size was calculated using PASS version 15 (PASS Software. NCSS, LLC. Kaysville, Utah, USA). Xie et al.'s previous research (23) among Chinese patients with coronary artery disease found that MACCE (including death from any cause, myocardial infarction, stroke, and ischemia-driven target-vessel revascularization) occurred in 2.66% of the personalized treatment group and 9.03% of the conventional treatment group within 6 months. According to their previous work and the experience at our clinical center, we assumed that MACCE rates in the GT and RT groups within 12 months were ~3.5 and 13%, respectively. We estimated that at least 182 patients in the GT group and 91 patients in the RT group would be needed to ensure a power of 80% to detect a significant difference in the primary endpoint using a two-tailed test, 5% type 1 error, and with a sampling ratio of 2:1 (GT:RT).

IBM SPSS version 21.0 (IBM Corp., Armonk, NY, USA) was used to perform statistical analysis. The analysis was conducted on the basis of the intention-to-treat principle. Categorical variables were reported as number and percentage. These were compared using chi-square or Fisher's exact-tests. For continuous variables, normally distributed data were presented as mean ± standard deviation and were compared with the Student *t*-test. Non-normally distributed data were presented as median (interquartile range) and were compared using the Mann–Whitney *U*-test. Kaplan–Meier curves were drawn using Prism version 7.0 to compare primary and secondary endpoints in a log-rank test (GraphPad Software, San Diego, CA, USA). Cox proportional hazards models were used to calculate hazard ratios with 95% confidence intervals. *P* < 0.05 was considered statistically significant.

## Results

### Baseline Characteristics

**Figure 1:**
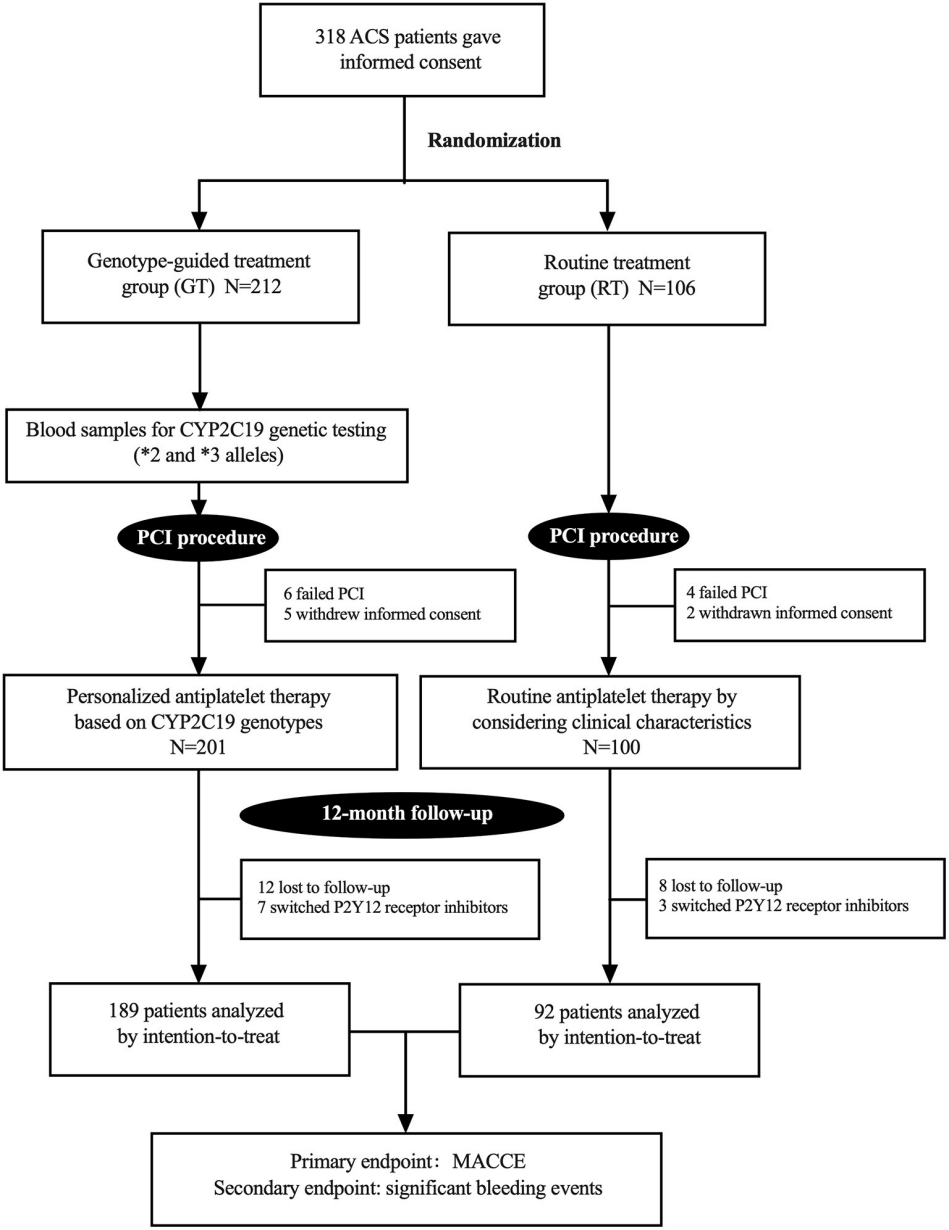
Study flow chart. ACS, acute coronary syndrome; PCI, percutaneous coronary intervention; MACCE, major adverse cardiovascular or cerebrovascular events.

From April 2019 to August 2019, 318 patients provided their informed consent and were randomly divided into a GT group (*n* = 212) and RT group (*n* = 106). Among them, 10 patients failed PCI and 7 patients were withdrawn from this study at their request. Finally, 301 patients were enrolled in the study, including 201 patients in the GT group and 100 in the RT group. The study procedure is shown in Figure 1. Among 301 enrolled patients, 75.1% were men, and the overall mean age was 59.7 ± 9.8 years. Of the total, 88.7% patients were diagnosed with unstable angina, 4.7% were diagnosed with STEMI, and 6.6% were diagnosed with non-STEMI. One hundred twelve (37.2%) patients had previous revascularization. Other characteristics were well balanced between the two groups (*P* > 0.05; Table 1). During the 12-month follow-up, 10 (3.3%) patients switched P2Y_12_ receptor inhibitors, 7 (3.5%) in the GT group and 3 (3.0%) in the RT group.

**Table 1:** Demographic and characteristics of the study population.

**Characteristics**	**All patients *n* = 301**	**RT *n* = 100**	**GT *n* = 201**	***P***
Age, years, mean (SD)	59.7 (9.8)	59.8 (10.4)	59.7 (9.6)	0.931
Male, *N* (%)	226 (75.1)	79 (79.0)	147 (73.1)	0.268
BMI, kg/m^2^^*^	25.8 (4.0)	25.8 (4.3)	25.7 (4.0)	0.775
Current smoker, *N* (%)	101 (33.6)	34 (34.0)	67 (33.3)	0.908
Current drinker, *N* (%)	119 (39.5)	42 (42.0)	77 (38.3)	0.537
Ethnicity, Han, *N* (%)	293 (97.3)	98 (98.0)	195 (97.0)	0.617
**Medical history**, ***N*** **(%)**				
Hypertension	193 (64.1)	66 (66.0)	127 (63.2)	0.631
Diabetes	113 (37.5)	38 (38.0)	75 (37.3)	0.908
Hyperlipidemia	205 (68.1)	67 (67.0)	138 (68.7)	0.771
Stroke	19 (6.3)	10 (10.0)	9 (4.5)	0.063
Peripheral arterial disease	4 (1.3)	1 (1.0)	3 (1.5)	0.725
Atrial fibrillation	1 (0.3)	0 (0)	1 (0.5)	0.480
**Previous revascularization**, ***N*** **(%)**				
PCI	105 (34.9)	39 (39.0)	66 (32.9)	0.291
CABG	7 (2.3)	3 (3.0)	4 (2.0)	0.584
**PCI indication**, ***N*** **(%)**				
STEMI	14 (4.7)	6 (6.0)	8 (4.0)	0.433
Non-STEMI	20 (6.6)	9 (9.0)	11 (5.5)	0.247
Unstable angina	267 (88.7)	85 (85.0)	182 (90.5)	0.152
**PCI type**, ***N*** **(%)**				
Drug-eluting stent	252 (83.7)	81 (81.0)	171 (85.1)	0.367
Balloon angioplasty	49 (16.3)	19 (19.0)	30 (14.9)	0.367
**Medical treatment**, ***N*** **(%)**				
Aspirin	297 (98.7)	99 (99.0)	198 (98.5)	0.725
Beta-blocker	183 (60.8)	60 (60.0)	123 (61.2)	0.842
ACEI and ARB	118 (39.2)	38 (38.0)	80 (39.8)	0.763
Statin	299 (99.3)	99 (99.0)	200 (99.5)	0.613
Calcium channel inhibitor	90 (29.9)	29 (29.0)	61 (30.3)	0.810
PPI	230 (76.4)	82 (82.0)	148 (73.6)	0.107

*RT, routine treatment group; GT, genotype-guided treatment group; BMI, body mass index; PCI, percutaneous coronary intervention; CABG, coronary-artery bypass grafting; STEMI, ST-segment elevation myocardial infarction; ACEI, angiotensin-converting enzyme inhibitor; ARB, angiotensin receptor blocker; PPI, proton pump inhibitor*.

* *The data were presented as median (interquartile range) and were calculated by Mann–Whitney U-test*.

### CYP2C19 Genotypes Distribution and P2Y_12_ Receptor Inhibitor Selection

Two hundred one patients in the GT group underwent CYP2C19 genotyping. Among them, 115 (57.2%) patients carried LOF alleles, including 78 (38.8%) who carried one LOF allele and 37 (18.4%) who carried two LOF alleles; 86 (42.8%) patients were non-carriers of LOF alleles. The details are shown in Table 2.

**Table 2:** CYP2C19 genotypes distribution and P2Y_12_ receptor inhibitors selection.

**Items**	**RT *n* = 100**	**GT *n* = 201**
**CYP2C19 genotypes**		
**LOF alleles carriers**, ***N*** **(%)**		
*2/*2	NA	20 (10.0)
*3/*3	NA	2 (1.0)
*2/*3	NA	15 (7.5)
*1/*2	NA	54 (26.9)
*1/*3	NA	24 (11.9)
**Non-LOF alleles carriers**, ***N*** **(%)**		
*1/*1	NA	86 (42.8)
**P2Y12 receptor inhibitors**		
Clopidogrel	75 (75.0)	86 (42.8)
Ticagrelor	25 (25.0)	115 (57.2)

*RT, routine treatment group; GT, genotype-guided treatment group; LOF, loss-of-function*.

**Figure 2:**
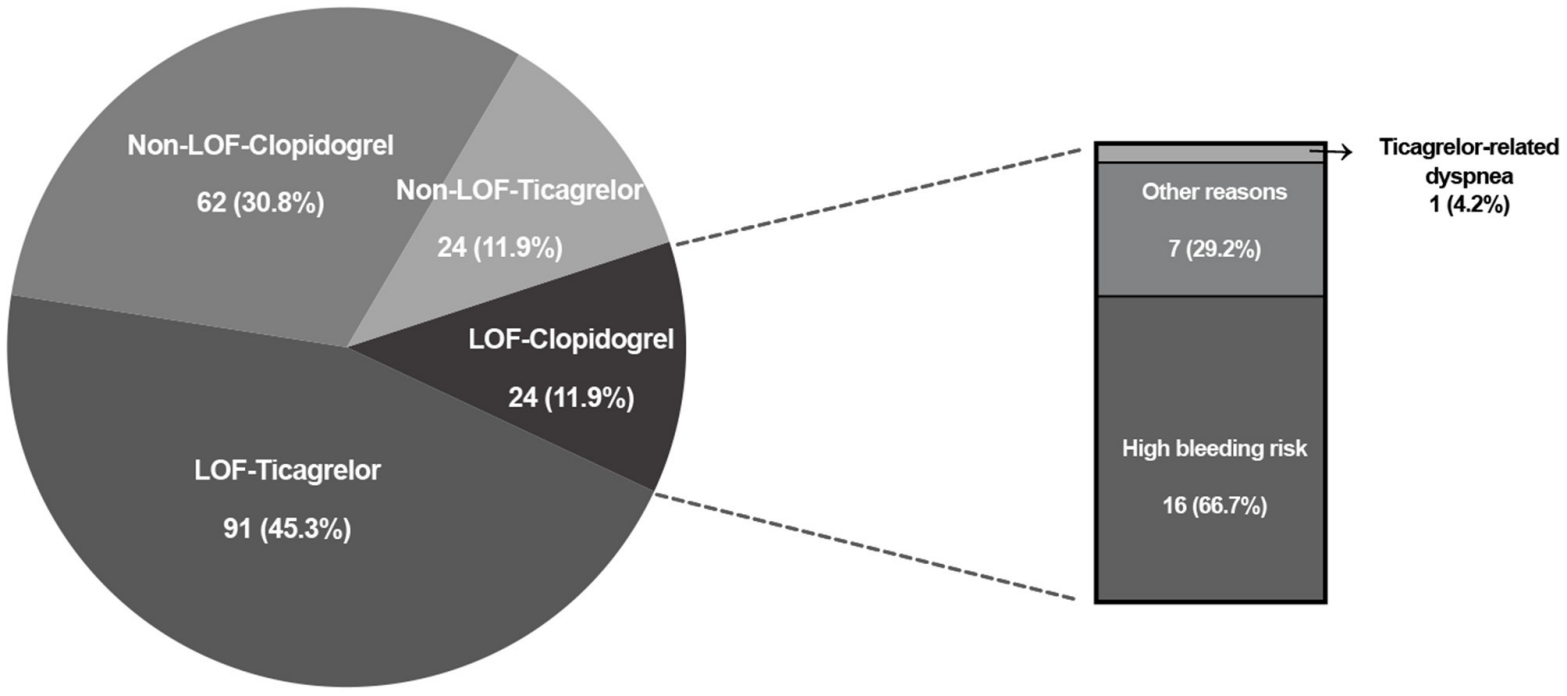
CYP2C19 LOF alleles distribution and P2Y_12_ receptor inhibitors selection in genotype-guided treatment group. LOF, loss-of-function.

According to clinical characteristics, 75 (75.0%) patients in the RT group were treated with clopidogrel. In the GT group, the frequency of clopidogrel treatment was 42.8%. Compared with the RT group, fewer patients used clopidogrel in the GT group (*P* < 0.001). Patients carrying LOF alleles were recommended treatment with ticagrelor; however, 24 LOF allele carriers were still using clopidogrel (Figure 2). The reasons for these patients not being prescribed ticagrelor included high bleeding risk (16/24), ticagrelor-related dyspnea (1/24), and other factors judged by cardiologists (7/24) (Figure 2). Of 86 non-carriers of LOF alleles, 62 used clopidogrel and 24 used ticagrelor.

### Outcomes

**Figure 3:**
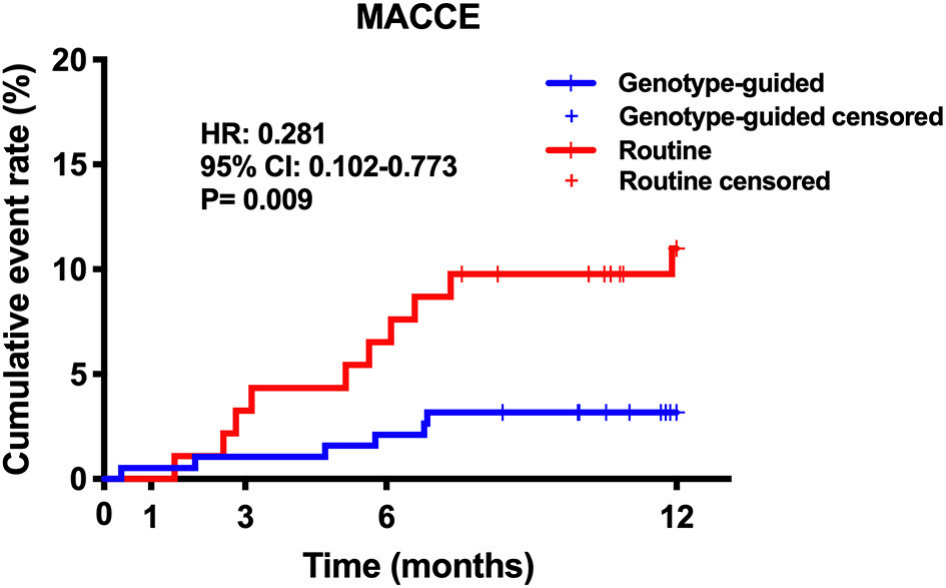
Kaplan–Meier curve of MACCE during 12-month follow-up. MACCE, major adverse cardiovascular or cerebrovascular events.

**Figure 4:**
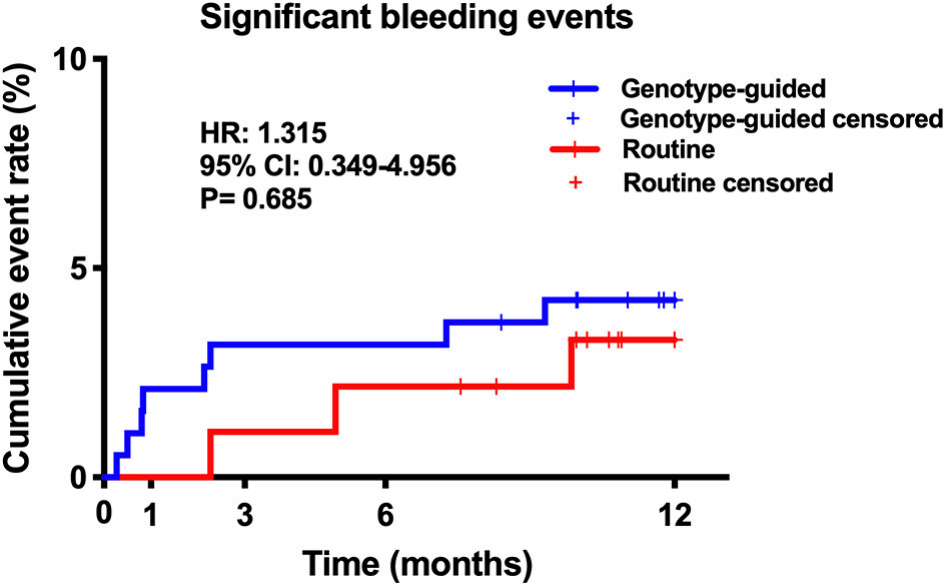
Kaplan–Meier curve of significant bleeding events during 12-month follow-up. Significant bleeding events: Bleeding Academic Research Consortium (BARC) class 2 or higher.

Twenty (6.6%) patients were lost to follow-up, 12 patients (6.0%) in the GT group and 8 (8.0%) in the RT group. No significant difference was found in the rate of loss to follow-up (*P* > 0.05). Within the 12-month follow-up, 16 (5.7%) patients experienced MACCE, 6 from the GT group and 10 from the RT group. The GT group showed lower rates of MACCE than the RT group (6/189 vs. 10/92, 3.2 vs. 10.9%, hazard ratio: 0.281, 95% confidence interval: 0.102–0.773, *P* = 0.009); the results are shown in Table 3 and Figure 3. Significant bleeding events occurred in 11 patients, 8 (4.2%) in the GT group and 3 (3.3%) in the RT group. There was no statistical difference in significant bleeding events between the two groups (hazard ratio: 1.315, 95% confidence interval: 0.349–4.956, *P* = 0.685); the results are given in Table 3 and Figure 4. Supplementary Table 1 shows the incidence of MACCE and significant bleeding events when patients were stratified by age and sex; all values of P for interaction were >0.05.

**Table 3:** Primary and safety endpoints during 12-month follow-up.

**Outcomes**	**RT (*n* = 92)**	**GT (*n* = 189)**	**Hazard ratio (95% CI)**	***P***
**MACCE**, ***N*** **(%)**	10 (10.9)	6 (3.2)	0.281 (0.102–0.773)	0.009
All-cause death	0 (0)	1 (0.5)		
Myocardial infarction	2 (2.2)	1 (0.5)		
Stroke	2 (2.2)	1 (0.5)		
Urgent coronary revascularization	8 (8.7)	3 (1.6)		
Stent thrombosis	6 (6.5)	4 (2.1)		
**Significant bleeding events**, ***N*** **(%) (BARC** **≥** **2)**	3 (3.3)	8 (4.2)	1.315 (0.349–4.956)	0.685

*RT, routine treatment group; GT, genotype-guided treatment group; MACCE, major adverse cardiovascular or cerebrovascular events; BARC, Bleeding Academic Research Consortium*.

## Discussion

To the best of our knowledge, this was the first randomized-controlled study to evaluate the effects of personalized pharmacogenomic antiplatelet therapy within a period of 12 months in the Chinese population. Our results showed that personalized antiplatelet therapy based on CYP2C19 genotypes could decrease the risk of MACCE within a 12-month period in patients with ACS undergoing PCI.

CYP2C19 genotypes varied among the patients enrolled in this study. The proportion of CYP2C19 LOF allele carriers (57.2% in the GT group) was similar to that in previous studies among Asian populations and was significantly higher than that in Western populations [~29.2% in Europe (13) and 30% in North America (24)]. Previous studies have reported that LOF allele carriers have a higher risk of adverse cardiovascular events than non-carriers among patients treated with clopidogrel (25, 26). Racial differences in CYP2C19 allele frequency suggest that a DAPT strategy in Asians needs to be considered prudently. Additionally, the proportion of patients with unstable angina among those undergoing PCI was 88.9% in this study, which was not consistent with other randomized clinical trials; this proportion was ~1.9% in a study by Notarangelo et al. (13) conducted in Italy, 38.6% in a multicenter study by Pereira et al. (27), and 72.0% in research by Xu et al. (28) in China. The reasons for this may be the differences in medical conditions and cardiovascular disease characteristics among countries and the limitation of a single-center trial in terms of representativeness compared with multicenter trials, as well as the expertise of the single clinical center in this study.

Our findings confirmed that personalized pharmacogenomic antiplatelet therapy could benefit Chinese patients with ACS after PCI. Our main findings are consistent with those of previous studies. Xie et al. (23) used data of Chinese patients and showed that personalized antiplatelet therapy according to CYP2C19 genotype in patients undergoing PCI decreased the incidence of major adverse cardiovascular events and the risk of 180-day stent thrombosis. This pharmacogenomic approach has also been reported to benefit Western populations, who have lower LOF allele frequencies. In the Pharmacogenetics of Clopidogrel in Acute Coronary Syndromes (PHARMCLO) trial (13) conducted in Italy, selecting P2Y12 receptor inhibitors by considering CYP2C19 genetic testing results was demonstrated to reduce ischemic events in patients undergoing PCI, and there were no differences between groups for major bleeding events. The Point-of-Care Genetic Testing for Personalization of Antiplatelet Treatment (RAPID GENE) study (14) in Canada reported benefits of CYP2C19 genetic testing in reducing high on-treatment platelet reactivity.

The latest Tailored Antiplatelet Initiation to Lessen Outcomes due to Decreased Clopidogrel Response After Percutaneous Coronary Intervention (TAILOR-PCI) trial (27) was a multicenter randomized study conducted in North America and Korea. The TAILOR-PCI trial found that major adverse cardiovascular events did not significantly decrease with CYP2C19 genotype-guided selection of P2Y12 inhibitor therapy at 12 months. Although the TAILOR-PCI trial did not meet its primary endpoint, the *P*-value was 0.06. The differences in enrolled patients at baseline may contribute to the difference in the main findings between our study and those of the TAILOR-PCI study. Compared with that trial, the present study had a higher proportion of patients with ACS (100 vs. 85%), previous revascularization (37 vs. 25%), diabetes (38 vs. 28%), and current smokers (34 vs. 25%), which are known ischemic risk factors (16). Implementing CYP2C19 genetic testing and treatment using more potent antiplatelet drugs might have greater benefits in high-risk populations (29). Of note, *post-hoc* analysis of the primary endpoint in the TAILOR-PCI trial showed that genotype-guided antiplatelet therapy could benefit patients undergoing PCI in reducing major adverse cardiovascular events at 3 months, suggesting that the genotype-guided strategy might be more important early after PCI. A similar result was not found in the present study, which might be caused by factors owing to the small number of participants and the primary endpoint. Further study with more participants is needed to identify the effect of this pharmacogenomic approach in the early post-PCI period.

CYP2C19 genotype was not the only factor taken into account in the selection of P2Y_12_ receptor inhibitors. Other clinical characteristics were also considered by cardiologists such as age, weight, diabetes, and ischemic and bleeding risks. In our study, 20.8% (24/115) of LOF allele carriers were treated with clopidogrel. Bleeding risk was the main reason (16/24) for cardiologists being reluctant to switch from clopidogrel to ticagrelor, which has been associated with more bleeding events than the former agent in East Asian populations. Our results demonstrated that personalized antiplatelet therapy in which CYP2C19 genotypes as well as clinical characteristics are considered might be a good approach that will help to strike a safe and effective balance between ischemic and bleeding events.

Clopidogrel remains the most widely used P2Y_12_ receptor inhibitor. In 2017, Jianan Li et al. (30) reported the utility of clopidogrel and ticagrelor in China. Their results showed that ~89.3% of patients with coronary artery disease undergoing PCI were treated with clopidogrel. In the present study, clopidogrel was used more frequently than ticagrelor in the RT group (75.0 vs. 25.0%). However, the frequency of clopidogrel treatment in European countries where ticagrelor and prasugrel are recommended as front-line antiplatelet drugs is lower than in China [e.g., ~50.7% in Italy (13)]. The higher frequency of clopidogrel use in East Asia indicates that CYP2C19 genotypes should be fully considered in the application of antiplatelet therapy in Asian populations.

CYP2C19 genotype-guided antiplatelet therapy has not been widely adopted. The black box warning of the US Food and Drug Administration recommends that CYP2C19 poor metabolizers should be avoided when using clopidogrel, but CYP2C19 genetic testing is not mandated (15). Similarly, PCI guidelines of the American College of Cardiology Foundation, American Heart Association, and the Society for Cardiovascular Angiography and Interventions recommend that CYP2C19 genetic testing be conducted in high-risk patients undergoing PCI, but not in all patients receiving PCI as routine treatment. Chinese guidelines for non-ST-segment elevation acute coronary syndrome also do not recommend routine genetic testing owing to a lack of clear evidence (31). To a certain extent, the present study findings may provide evidence for CYP2C19 genetic testing among Asian patients undergoing PCI.

The delay in obtaining the results of genetic testing also impedes the clinical utility of CYP2C19 genetic testing. Generally, the time to obtaining such results varies from 2 to 4 working days after sample collection (32), at which point many patients have already begun P2Y12 inhibitor therapy. Although these patients are still hospitalized, cardiologists are often reluctant to switch existing antiplatelet therapy according to the delayed genetic testing results (33). However, with advances in genetic testing technology, the time is shortening between genetic tests being administered and physicians receiving the results. In Lee et al.'s study (34), the median time to receiving genetic testing results was only 1 day. Moreover, rapid CYP2C19 genotyping, which usually yields results within 1–2 h, is feasible in the clinic and is associated with fewer adverse events in patients with ACS (14). The development of rapid genotyping technology may play an important role in promoting the clinical utility of personalized CYP2C19 genotype-guided antiplatelet therapy.

A pharmacogenomic approach may have a key role in the de-escalation of antiplatelet therapy. The CYP2C19 Genotype-Guided Antiplatelet Therapy in ST-Segment Elevation Myocardial Infarction Patients—Patient Outcome after Primary PCI (POPular Genetics) trial (35) conducted in Europe compared CYP2C19 genotype-guided antiplatelet therapy (LOF allele carriers used ticagrelor or prasugrel and non-carriers used clopidogrel) with standard treatment (mainly ticagrelor or prasugrel). The results showed that de-escalation of CYP2C19 genotype-guided therapy decreased minor and major bleeding events (9.8 vs. 12.5%, hazard ratio: 0.78, 95% confidence interval: 0.61–0.98, *P* = 0.04) without increasing thrombotic risk (2.7 vs. 3.3%, hazard ratio: 0.83, 95% confidence interval: 0.53–1.31). The 2020 European Society of Cardiology Guidelines for the management of ACS in patients presenting without persistent ST-segment elevation recommend that CYP2C19 genotype-guided de-escalation of antiplatelet therapy might be considered as an alternative DAPT strategy(IIb, A) (36). All of the above studies demonstrated that personalized antiplatelet therapy based on CYP2C19 genotypes might be a valuable strategy to optimize DAPT in patients with ACS and warrants further research in the future.

Our study has some limitations. First, CYP2C19 allele 17, the gain-of-function allele, was not detected in our study. However, CYP2C19 allele 17 is reported to have a low mutation rate in Chinese people [~0.8% (37)] and it does not affect the identification of LOF allele carriers. Second, because prasugrel is not licensed in China, ticagrelor was the only alternative P2Y_12_ receptor inhibitor in this study. Third, this trial was open-label, which might create potential bias; however, blinding was used in the adjudication and analysis of all events. Fourth, the small number of participants and primary endpoint events were important limitations in this study. However, a larger number of patients than the calculated sample size could not be enrolled for ethical and economic reasons. Fifth, because no non-inferiority assumption was included for the risk of bleeding events when calculating the sample size, the test power comparing bleeding risk between two groups was insufficient and thus requires further investigation. Sixth, this was a single-center trial, and most participants were Han ethnicity; multicenter studies of more ethnically diverse groups are needed to confirm our conclusions in East Asian populations. Finally, the analysis was conducted according to the intention-to-treat principle. The reasons involved in patients switching P2Y_12_ inhibitors were not further studied. However, only 3.3% of patients switched P2Y_12_ inhibitors. This proportion was acceptable and might not influence the main findings.

## Conclusions

Personalized antiplatelet therapy that is based on CYP2C19 genotypes decreased MACCE within 12 months in Chinese patients with ACS undergoing PCI.

## Data Availability Statement

The raw data supporting the conclusions of this article will be made available by the authors, without undue reservation.

## Ethics Statement

The studies involving human participants were reviewed and approved by Ethics Committee of Beijing Anzhen Hospital. The patients/participants provided their written informed consent to participate in this study.

## Author Contributions

XS and YLi: conception and design and administrative support. WL, ZF, ZZ, and YLv: determination of clinical events. YiZ, YuZ, RZ, BL, JH, JY, and YW: collection and upload of data. YuZ, YiZ, JH, and BL: data analysis and interpretation. XS, YuZ, and YiZ: manuscript writing. All authors contributed to the article and approved the submitted version.

## Conflict of Interest

The authors declare that the research was conducted in the absence of any commercial or financial relationships that could be construed as a potential conflict of interest.
