# Supplementary material for: Personalized Antiplatelet Therapy Based on CYP2C19 Genotypes in Chinese ACS Patients Undergoing PCI: A Randomized Controlled Trial
Source: Front Cardiovasc Med. 2021 Jun 16;8:676954. doi: 10.3389/fcvm.2021.676954 (PMC8242578; doi:10.3389/fcvm.2021.676954)
Supplement: Supplementary file 1 [file Table_1.docx]

Table S1. The incidence of MACCE and significant bleeding events during 12-month follow-up was stratified by age and gender.

| Variables | MACCE | | | |  | Significant bleeding events | | | |
| --- | --- | --- | --- | --- | --- | --- | --- | --- | --- |
|  | RT  (n=92) | GT  (n=189) | HR  (95% CI) | *P* for  interaction |  | RT  (n=92) | GT  (n=189) | HR  (95% CI) | *P* for  interaction |
| **Age, years** |  |  |  | 0.339 |  |  |  |  | 0.514 |
| ≥65 | 3/35 (8.6) | 3/64 (4.7) | 0.523 (0.106-2.593) |  |  | 1/35 (2.9) | 1/64 (1.6) | 0.514 (0.032-8.234) |  |
| <65 | 7/57 (12.3) | 3/125 (2.4) | 0.187 (0.048-0.725) |  |  | 2/57 (3.5) | 7/125 (5.6) | 1.587 (0.330-7.646) |  |
| **Sex** |  |  |  | 0.330 |  |  |  |  | 0.660 |
| Male | 8/72 (11.1) | 3/139 (2.2) | 0.186 (0.049-0.702) |  |  | 2/72 (2.8) | 6/139 (4.3) | 1.540 (0.311-7.639) |  |
| Female | 2/20 (10.0) | 3/50 (6.0) | 0.573 (0.096-3.429) |  |  | 1/20 (5.0) | 2/50 (4.0) | 0.800 (0.072-8.819) |  |

RT: routine treatment group; GT: genotype-guided treatment group; MACCE: major adverse cardiovascular or cerebrovascular events.
